# Rationale, design and baseline characteristics of participants in the OCEANIC-STROKE trial of FXIa inhibition for secondary stroke prevention

**DOI:** 10.1093/esj/aakaf017

**Published:** 2026-01-01

**Authors:** Mukul Sharma, Qiang Dong, Teruyuki Hirano, Scott E Kasner, Jeffrey Saver, Jaime Masjuan, Andrew M Demchuk, Charlotte Cordonnier, Daniel Bereczki, Georgios Tsivgoulis, Roland Veltkamp, Ivan Staikov, Hee-Joon Bae, Bruce C V Campbell, Andrea Zini, I-Hui Lee, Sebastian Ameriso, Martin Kovar, Robert Mikulik, Robin Lemmens, José M Ferro, Thompson Robinson, Hanne Christensen, Serefnur Ozturk, Ronen R Leker, Peter Turcani, Agnieszka Slowik, Pablo Amaya, Fan Kee Hoo, Gian Marco De Marchis, Michael Knoflach, Pillai N Sylaja, Jukka Putaala, Jonathan M Coutinho, H Bart van der Worp, Evija Miglane, Vaidas Matijosaitis, Arne G Lindgren, Gisele Sampaio Silva, Else Charlotte Sandset, Saule Tleubergenovna Turuspekova, Raed A Joundi, Karleen Schulze, Olga Shestakovska, Jennifer Gilbride, Shrikant I Bangdiwala, Lizhen Xu, Eva Muehlhofer, Pablo Colorado, Hardi Mundl, Lars Keller, Ashkan Shoamanesh

**Affiliations:** Population Health Research Institute, Hamilton Health Sciences, Hamilton, Canada; McMaster University, Department of Medicine, McMaster University, Hamilton, Canada; Department of Neurology, Huashan Hospital, Fudan University, Shanghai, China; Department of Stroke and Cerebrovascular Medicine, Kyorin University, Tokyo, Japan; Department of Neurology Perelman School of Medicine, University of Pennsylvania, Philadelphia, USA; Department of Neurology and Comprehensive Stroke Center, David Geffen School of Medicine at UCLA, Los Angeles, USA; Neurology Department, Hospital Universitario Ramón y Cajal, Madrid, Spain; Departments of Clinical Neurosciences and Radiology, Hotchkiss Brain Institute, Cumming School of Medicine, University of Calgary, Calgary, Canada; Department of Neurology and Stroke Center, Lille University Hospital Center, Lille, France; Department of Neurology, Semmelweis University, Budapest, Hungary; Second Department of Neurology, National & Kapodistrian University of Athens, School of Medicine, Athens, Greece; Department of Neurology and Epileptology, Alfried Krupp Krankenhaus, Essen, Germany; Department of Neurology and Sleep Medicine, Acibadem City Clinic University Hospital Tokuda, Sofia, Bulgaria; Department of Neurology, Seoul National University College of Medicine, Seoul National University Bundang Hospital, Seongnam, South Korea; Department of Medicine and Neurology, Royal Melbourne Hospital, University of Melborne, Parkville, Victoria, Australia; Department of Neurology and Stroke Center, Maggiore Hospital, IRCCS Istituto delle Scienze Neurologiche di Bologna, AUSL Bologna, Bologna, Italy; Department of Neurology, Taipei Veterans General Hospital, Taipei City, Taiwan; Section of Vascular Neurology, Institute for Neurological Research – FLENI, Buenos Aires, Argentina; Department of Neurology, Nemocnice Na Homolce, Prague, Czechia; International Clinical Research Center, St. Ann's University Hospital, Brno, Czechia; Department of Neurosciences, Experimental Neurology, KU Leuven, Leuven, Belgium; Department of Neurology, University Hospitals Leuven, Leuven, Belgium; Department of Neurology, Faculdade de Medicina da Universidade de Lisboa, Lisbon, Portugal; Department of Cardiovascular Sciences, University of Leicester, Leicester, UK; Department of Clinical Medicine, Copenhagen University Hospital, Copenhagen, Denmark; Department of Neurology, Selcuk University, Konya, Turkey; Department of Neurology, Hadassah-Hebrew University Medical Center, Jerusalem, Israel; 1st Department of Neurology, Comenius University, Bratislava, Slovakia; Clinical Department of Neurology, Szpital Uniwersytecki w Krakowie, Krakow, Poland; Neurology Department, Fundacion Valle del Lili University Hospital, Cali, Columbia; Department of Neurology, Universiti Putra Malaysia, Selangor, Malaysia; Department of Neurology, University Research and Teaching Hospital, HOCH-Kantonssspital St. Gallen, St. Gallen, Switzerland; Department of Neurology, Medical University Innsbruck, Innsbruck, Austria; Department of Neurology, Sree Chitra Tirunal Institute for Medical Sciences and Technology, Thiruvananthapuram, India; Neurocenter, Helsinki University Hospital, Helsinki, Finland; Department of Neurology, Amsterdam University Medical Centers, Amsterdam, Netherlands; Department of Neurology and Neurosurgery, Brain Center, University Medical Center Utrecht, Utrecht, Netherlands; Department of Neurology and Neurosurgery, P. Stradins Clinical University Hospital, Riga, Latvia; Riga Stradins University, Department of Neurology and Neurosurgery, Riga, Latvia; Department of Neurology, Lithuanian University of Health Sciences, Kaunas, Lithuania; Department of Neurology, Skåne University Hospital, Lund, Sweden; Department of Clinical Sciences Lund, Neurology, Lund University, Lund, Sweden; Department of Neurology, Universidade Federal de São Paulo-UNIFESP and Hospital Israelita Albert Einstein, Sao Paulo, Brazil; Department of Neurology, Oslo University Hospital, Oslo, Noway; Department of Nervous Diseases, Asfendiyarov Kazakh National Medical University, Almaty, Kazakhstan; Population Health Research Institute, Hamilton Health Sciences, Hamilton, Canada; McMaster University, Department of Medicine, McMaster University, Hamilton, Canada; Population Health Research Institute, Hamilton Health Sciences, Hamilton, Canada; McMaster University, Department of Medicine, McMaster University, Hamilton, Canada; Population Health Research Institute, Hamilton Health Sciences, Hamilton, Canada; McMaster University, Department of Medicine, McMaster University, Hamilton, Canada; Bayer AG, Wuppertal, Germany; Population Health Research Institute, Hamilton Health Sciences, Hamilton, Canada; McMaster University, Department of Medicine, McMaster University, Hamilton, Canada; Population Health Research Institute, Hamilton Health Sciences, Hamilton, Canada; McMaster University, Department of Medicine, McMaster University, Hamilton, Canada; Bayer AG, Wuppertal, Germany; Bayer AG, Wuppertal, Germany; Bayer AG, Wuppertal, Germany; Bayer AG, Wuppertal, Germany; Population Health Research Institute, Hamilton Health Sciences, Hamilton, Canada; McMaster University, Department of Medicine, McMaster University, Hamilton, Canada

**Keywords:** stroke, prevention, randomised trial, factor XI, asundexian

## Abstract

**Introduction:**

Genetic deficiency of factor XI is associated with a reduced risk of ischemic stroke. Asundexian is a direct inhibitor of activated factor XIa (FXIa) with a low risk of bleeding in early trials. We seek to determine its efficacy and safety combined with antiplatelet therapy for prevention of ischemic stroke.

**Patients and methods:**

Oral faCtor Eleven A iNhibitor asundexian as novel antithrombotiC (OCEANIC-STROKE) is a placebo-controlled, double-blind, event-driven randomised trial including participants with stroke (NIHSS ≤ 15) or high-risk TIA (ABCD^2^ 6 or 7) within 72 h of onset. Participants had at least one of the following: atherosclerosis of extra- or intracranial vessels, a medical history of atherosclerosis or an imaged acute non-lacunar infarct. We excluded sources of stroke requiring anticoagulation and active non-trivial bleeding other than hemorrhagic infarction (HI 1 or 2). Participants received asundexian 50 mg daily or placebo stratified by planned concurrent antiplatelet therapy (single vs dual). The primary endpoint is time to ischemic stroke. We present baseline characteristics as of 5 June 2025.

**Results:**

Between January 2023 and February 2025, we randomised 12,327 participants. Participants were 67% male with a mean (SD) age of 68 (11) years. Ischemic stroke was the index event for 95% of whom 27.4% had thrombolysis and/or mechanical thrombectomy. By TOAST classification, 43% of index strokes were LAA, 22% small vessel disease, 30% undetermined and 2% cardioembolic. Dual antiplatelets were planned in 63% as standard initial treatment. Trial completion is anticipated in October 2025.

**Conclusion:**

OCEANIC-STROKE will be the first completed trial of FXIa inhibition for prevention of stroke after non-cardioembolic stroke or TIA.

**Trial registration:**

ClinicalTrials.gov (NCT05686070).

## Graphical Abstract

**Graphical Abstract:**
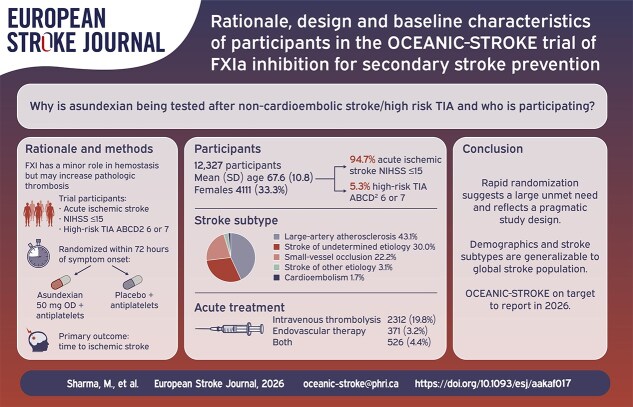

## Introduction

Current antithrombotic options for secondary prevention after non-cardioembolic stroke have limited efficacy and a risk of haemorrhage. Most ischemic strokes are not cardioembolic, with cardiac emboli accounting for only 25%–30% of the total.^1^ Individuals with a non-cardioembolic stroke or TIA have a risk of stroke recurrence of up to 12% at 1 year.^2^ Anticoagulation in the setting of atrial fibrillation (AF) is very effective and is associated with an approximately 2/3 reduction in the risk of stroke.^3^ This advance stands in distinction to the current standard antithrombotic treatment for non-cardioembolic stroke, which consists of short-term dual antiplatelets for minor stroke and TIA, and single antiplatelets for moderate–severe stroke, followed by long-term single antiplatelet treatment, usually aspirin or clopidogrel.^4^ Dual antiplatelet therapy, combining aspirin with clopidogrel or ticagrelor, reduces the risk of stroke recurrence over aspirin alone in patients with minor ischemic stroke or TIA, but is associated with increased risk of haemorrhage, and use is restricted to a brief period after an acute cerebrovascular event.^5–7^ Long-term use of clopidogrel and aspirin for secondary prevention has not reduced stroke recurrence over single antiplatelet therapy and has been associated with increased bleeding and mortality.^8^^,^^9^ Clopidogrel is a prodrug that is converted to its active form and has reduced efficacy in people with loss-of-function alleles in CYP2C19, a potentially significant limitation in populations with a high prevalence of poor metabolisers.^10^^,^^11^ Ticagrelor, which does not require conversion to an active form has evidence to support short-term use in combination with aspirin, but there is no evidence to support long-term use.^7^ Dual antiplatelet therapy with cilostazol, a phosphodiesterase inhibitor, may have some benefit compared with monotherapy for long-term treatment, but evidence for benefit for stroke recurrence soon after a stroke or TIA is lacking, and the current evidence has not resulted in international adoption of cilostazol.^4^^,^^12^ Dipyridamole, another phosphodiesterase inhibitor, is used in combination with aspirin, though evidence suggests this combination is not superior to clopidogrel monotherapy.^13^ There is an unmet need for antithrombotic strategies that reduce the risk of stroke occurrence after a non-cardioembolic stroke or TIA without increasing the risk of haemorrhage.

Thrombi that result in cerebrovascular occlusion may arise from multiple sources with varying proportions of platelets, red blood cells, and fibrin.^14^ Further, platelets and fibrin may interact to promote thrombus formation, and optimal antithrombotic therapy may require targeting both platelet activation and thrombin generation.^15^ The combination of low-dose rivaroxaban (a factor Xa inhibitor) with aspirin was associated with a reduced occurrence of major adverse cardiovascular events compared with aspirin. The risk of ischemic stroke was significantly reduced without an increase in hemorrhagic stroke, but there was an increase in systemic, mostly gastrointestinal, haemorrhage with this combination.^16^^,^^17^

**Figure 1:**
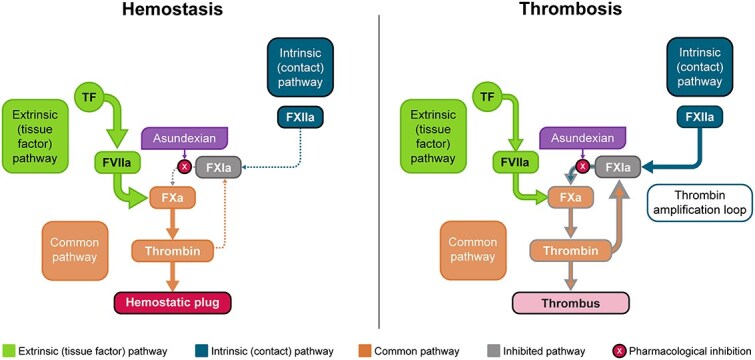
FXI(a) in the coagulation cascade. The extrinsic (tissue factor) pathway (light green) is activated after vascular damage, forming a thrombin burst to build a hemostatic plug at the site of injury and thereby stop any bleeding and ensure haemostasis. The intrinsic (contact) pathway (dark blue) is activated by negatively charged surfaces from for example, activated cells, leading to FXI activation and clot formation within the vessel. Thrombin itself activates FXI via the amplification loop to generate a secondary burst of thrombin, allowing an initial clot to grow and obstruct the vessel lumen. While both pathways of thrombus formation are inhibited by FX inhibitors, inhibiting FXIa maintains the ability to form a clot at the specific site of vessel wall injury and haemostasis is relatively independent of FXIa. Dashed arrows indicate minimal involvement of FXIa in haemostasis.

Factor XI, a zymogen, is converted to its activated protease form, FXIa, by intrinsic coagulation pathway activation. Preclinical, epidemiologic, and phase II trial evidence suggests that FXIa has a much stronger association with pathologic thrombi than with haemostasis, and inhibition of FXIa may reduce the occurrence of ischemic stroke without increasing bleeding. (Figure 1) Genetically determined FXI levels correlate with the risk of ischemic stroke in human populations. Deficiency of FXI is associated with a lower risk of ischemic stroke without an increased risk of intracerebral haemorrhage.^18–20^ Conversely, those with high levels of FXI have a higher risk of ischemic stroke.^21^ Spontaneous bleeding in FXI deficiency is rare and occurs mainly in tissues with high intrinsic fibrinolytic activity such as the nasopharynx and the urinary system.^22^ The potential to reduce ischemic stroke without increasing haemorrhage makes FXI an attractive therapeutic target for secondary stroke prevention.

Asundexian is a direct, potent small molecule inhibitor of FXIa that reduces the occurrence and size of arterial and venous thrombi in animal models without affecting bleeding time, alone or in combination with dual antiplatelet therapy (DAPT).^23^ Excretion is mainly faecal and dosing is not dependent on renal function.^24^ More than 4000 participants were studied in phase II trials that included people with acute coronary syndromes, AF, and acute ischemic stroke. In dose-finding trials in participants with AF or acute coronary syndrome, asundexian 50 mg once daily was associated with >90% inhibition of FXIa at peak and trough, significantly less bleeding than apixaban and no statistically significant increase in major bleeding compared with placebo, when combined with antiplatelets.^25^^,^^26^

In a randomised, placebo-controlled, double-blind, dose-finding trial of asundexian combined with antiplatelets in 1808 participants with acute non-cardioembolic ischemic stroke, there was no statistically significant increase in major or clinically relevant non-major bleeding.^27^ The primary efficacy outcome of clinical stroke and MRI-determined covert infarct did not show a dose–response effect with asundexian, possibly due to the absence of an effect on covert infarcts. Symptomatic ischemic stroke was numerically, but not significantly, less common in participants assigned asundexian 50 mg compared with placebo. In exploratory analyses, there was a significant reduction in the occurrence of symptomatic ischemic stroke or TIA, that was further reduced in those with intra- or extracranial atherosclerosis of any degree or location in participants assigned asundexian 50 mg compared with placebo. There was no statistically significant increase in hemorrhagic transformation over placebo of the index ischemic stroke or new microhaemorrhages on study MRI (performed in a subset of 1746 participants) after study drug initiation in those assigned asundexian.^28^ Pooled analysis of bleeding in phase II trials with a combined total of more than 500 bleeding events, showed no significant association between asundexian exposure and major bleeding.^29^

## Patients and methods

### Overview

The Oral faCtor Eleven A iNhibitor asundexian as novel antithrombotiC (OCEANIC-STROKE) trial is an international, phase III, randomised, double-blind, placebo-controlled, event-driven clinical trial examining the efficacy and safety of asundexian 50 mg daily in participants with acute non-cardioembolic stroke or high-risk TIA.

**Table 1:** Key inclusion criteria

**Participant and disease characteristics**	
Participants who have an acute onset of neurological deficit attributed to non- cardioembolic focal brain ischemia due to either:	
Non-cardioembolic ischemic stroke with NIHSS ≤ 15 at randomisation ***AND*** Persistent signs and symptoms of stroke lasting for *>* 24 h ***OR*** Acute ischemic brain infarction documented by MRI (diffusion weight imaging), standard CT or perfusion CT that could account for the clinical presentation.	High-risk TIA with complete resolution of symptoms within < 24 h ***AND*** an ABCD^2^ score = 6 or 7 with negative neuroimaging (CT or MRI) for acute ischemia
**Additional criteria**	
All participants must have *at least one* of the following criteria a–c: Cerebrovascular atherosclerosis defined as vascular imaging (CTA, MRA, ultrasound and digital subtraction angiography) showing atherosclerotic plaque involving intracranial or extracranial cerebral arteries or the aortic arch^a^ Medical history of atherosclerosis: CAD or AMI with documented coronary atherosclerotic disease, prior CABG, or prior PCI PAD requiring previous bypass surgery, or percutaneous transluminal angioplasty revascularisation, limb or foot amputation for arterial vascular disease (ie, excludes trauma), OR history of intermittent claudication and one or more of the following: (1) an ankle/arm blood pressure (BP) ratio < 0.90, or (2) documented peripheral artery stenosis Carotid stenosis ≥50% or previous carotid revascularisation Documented aortic plaque Brain imaging demonstrating an acute non-lacunar infarct (CT, CT perfusion or DWI MRI) defined as cortical location and/or size > 20 mm for DWI and >15 mm for CT. If no brain infarct is documented prior to randomisation (ie, clinical diagnosis of stroke or TIA with negative imaging) at least one of the following needs to be present that is not otherwise explainable and is related to the acute ischemic stroke/TIA event: motor deficits, speech deficits (aphasia/dysarthria), visual deficits (hemianopsia) and/or neglect. Thus, patients with isolated dizziness/vertigo or isolated numbness are not eligible. Imaging of brain (CT or MRI) prior to randomisation ruling out hemorrhagic stroke or another pathology that could explain symptoms (eg, brain tumour, abscess)	
Plan for secondary prevention of stroke/TIA with single or dual antiplatelet therapy including aspirin, clopidogrel, ticagrelor, prasugrel, cilostazol and dipyridamole and in line with local guidelines. Able to be randomised within 72 h after the onset of symptoms of the index event (or after patients were last known to be without symptoms in case of wake-up stroke). NOTE: In case of endovascular therapy (mechanical thrombectomy) and/or thrombolysis, randomisation can only occur > 24 h after endovascular therapy and in case of thrombolysis only after 24 h and standard clinical imaging has been performed post thrombolysis to exclude haemorrhage.	

^a^Plaque need not be causative or stenotic but intruding into lumen.

### Study population

Key inclusion criteria are listed in Table 1. Adults with non-cardioembolic ischemic stroke with a NIHSS ≤ 15 or high-risk TIA (defined as ABCD^2^ scores 6 or 7) who could be randomised within 72 h of symptom onset were included after informed consent was obtained. The range of NIHSS eligible for inclusion was consistent with the scores established as likely safe in the Proper Dosing and safety of the Oral FXIa Inhibitor BAY 2433334 in Patients Following Acute Noncardioembolic Stroke (PACIFIC-STROKE phase II trial of asundexian.^27^ ABCD^2^ scores of 6 or 7 ensured that participants with TIA were at high risk for recurrent stroke as defined by the validation study of this score.^30^ All participants were required to have at least one of the following: (1) cerebrovascular imaging showing evidence of atherosclerosis at any location from the aortic arch to the intracranial vessels; (2) a history of atherosclerosis including coronary artery disease, peripheral vascular disease, asymptomatic carotid atherosclerosis or previous carotid revascularisation and (3) brain imaging demonstrating an acute non-lacunar infarct. Participants with lacunar infarcts were permitted provided they met one of the first two requirements. Participants were required to have a plan for antiplatelet treatment, either single or dual consistent with local practice at the time of randomisation.

Table 2 shows key exclusion criteria. Potential participants with AF or other types of stroke requiring anticoagulation were excluded as were those with active non-trivial bleeding. Clinical brain imaging was required prior to randomisation, and we excluded those with hemorrhagic transformation of the index stroke event resulting in parenchymal hematoma (PH1 or PH2 by the Heidelberg classification).^31^ Asymptomatic hemorrhagic transformation consisting of petechial haemorrhage (HI1 and HI2 by the Heidelberg classification), asymptomatic chronic macrohaemorrhages, cerebral microbleeds and superficial siderosis was permitted. A history of nontraumatic intracranial bleeding was an exclusion. Strokes following procedures or due to other rare causes (eg, cerebrovascular dissection, bacterial endocarditis) were excluded. Renal dysfunction was not an exclusion criterion, apart from a current or anticipated requirement for dialysis within 12 months after trial entry. A requirement for ongoing therapeutic anticoagulation, long-term non-steroidal anti-inflammatory drugs or strong inhibitors or inducers of P-glycoprotein and CYP3A4 were exclusions. Carotid revascularisation was not an exclusion criterion, as recurrent stroke may occur in the period between the index event and the procedure, and there is the potential for benefit in this population.

**Table 2:** Key exclusion criteria


Recent ischemic stroke within 7 days before index event Stroke (index event) following procedures (eg, Transcatheter aortic valve implantation (TAVI), coronary artery bypass grafting (CABG) or strokes due to other rare causes (eg, bacterial endocarditis, vertebral artery dissections) Known premorbid (before index event) mRS ≥ 4 History of atrial fibrillation/flutter, left ventricular thrombus, mechanical valve or other cardioembolic source of stroke requiring anticoagulation Sustained uncontrolled hypertension after index stroke/TIA event Known vascular malformation of the brain with high risk for bleeding (except isolated cavernoma, aneurysm treated and secured, or aneurysm with diameter < 5 mm) Active non-trivial bleeding (including PH1 or PH2 hemorrhagic transformation of the index stroke event, if known before randomisation); known chronic bleeding disorder (eg, von Willebrand disease); history of non-traumatic intracranial haemorrhage (does not include cerebral microbleeds or asymptomatic hemorrhagic transformation of an ischemic stroke); other non-traumatic major bleeding or clinically significant gastrointestinal bleeding within last 6 months before randomisation Known significant liver disease (eg, acute hepatitis, chronic active hepatitis, cirrhosis, or signs of coagulopathy) or known hepatic insufficiency classified as Child-Pugh B or C at randomisation End stage renal disease requiring dialysis or expected to be started on dialysis within the next 12 months Major surgery during the last 30 days prior to randomisation Known allergy, intolerance or hypersensitivity to the study intervention (asundexian or excipients) Concomitant use, planned use or anticipated need for Oral anticoagulation Full dose and/or long-term anticoagulation therapy with heparin/LMWH during study conduct Chronic (more than 4 weeks continuous) therapy with NSAIDs during the study conduct Concomitant use of combined P-gp and strong CYP3A4 inducers, eg, carbamazepine, St John’s wort, as well as within 14 days (or at least five half-lives of the active substance, whichever is longer) before randomisation Concomitant use of combined P-gp and strong CYP3A4 inhibitors for example, human immunodeficiency virus protease inhibitors, systemically used azole antimycotic agents (eg, ketoconazole), clarithromycin, nefazodone, as well as within 14 days (or at least five half-lives of the active substance, whichever is longer) before randomisation Herbal or traditional medicine, and/or supplements with known anticoagulant and/or antiplatelet effect. Known current alcohol and/or illicit drug abuse that may interfere with the participant’s safety and/or compliance at the discretion of the investigator

Sites were provided with training on the scientific importance of including a representative population of people with stroke, with special efforts made to increase the inclusion of women and other underrepresented groups under the guidance of a Diversity and Inclusion Subcommittee of the Steering Committee. Sites were provided with tools to aid in these efforts. Trial population characteristics were monitored at the national and site level and regular feedback was provided to sites on the proportions of participants in each group and their performance relative to targets.

### Study treatments and visits

**Figure 2:**
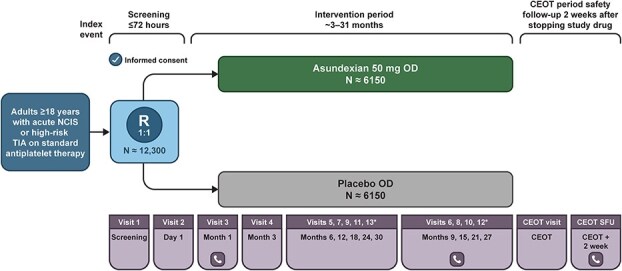
*Study design overview.*— 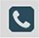 phone visit. Abbreviations: CEOT = common end of treatment; OD = once daily; R = randomisation; SFU = safety follow-up; W = weeks. ^*^ If applicable, visits will continue after month 30 in the same way as before until CEOT visit. Day 1 is the day of randomisation.

Following informed consent, participants were randomised 1:1 to asundexian 50 mg once daily or matching placebo using a central interactive randomisation system (see Figure 2). The randomisation used a fixed block size and was generated by an independent statistician with no other involvement in the trial. Randomisation was stratified by the planned antiplatelet treatment at the time of randomisation: single vs dual. Sites were asked to administer the first dose of study medication within 4 h of randomisation. Asundexian or matching placebo was continued through the treatment period lasting a minimum of 3 months and an anticipated maximum of 30 months. The intervention was administered orally as a whole tablet or crushed after the sponsor confirmed comparable pharmacokinetics of crushed administration compared with intact tablets. The protocol modification was made on 29 November 2023 by which point 5122 participants had been randomised. Participants, investigators and study personnel were masked to treatment assignment. Investigators were provided with guidance in the protocol on the management of haemorrhage and periprocedural issues occurring in participants taking study medication. We stopped study medication in participants diagnosed with AF during the study and recommended initiating anticoagulation consistent with local guidelines. Study visits occurred every 3 months during the treatment period and concluded with a common end of treatment (CEOT) visit when the required number of primary endpoints had been accumulated. A final safety visit was conducted 2 weeks after the CEOT visit for participants on treatment at their CEOT visit. Participants who discontinued study intervention were followed for efficacy and safety events unless there was a withdrawal of consent.

### Study objectives and endpoints

The primary efficacy endpoint is time to the first occurrence of ischemic stroke and the primary safety endpoint is major bleeding as defined by the International Society of Thrombosis and Haemostasis (Table 3 and Tables S1 and S2).^32^ A new ischemic stroke was considered present if either of the following were present: (1) a new focal neurological deficit persisting for ≥24 h not attributable to a non-ischemic cause or (2) worsening of an existing focal neurological deficit attributable to a new infarction or extension of the previous infarction in the same vascular territory, based on persisting symptoms/signs or imaging evidence of infarction with no evidence of a non-ischemic aetiology. Efficacy and safety endpoints were adjudicated and classified by trained adjudicators, blinded to treatment allocation using standard study definitions. Secondary endpoints included all strokes (combining ischemic and hemorrhagic stroke), hemorrhagic and disabling strokes. Exploratory endpoints included the modified Rankin scale as well as health-related quality of life measured by the European Quality of Life Group 5 Dimension questionnaire. An exploratory analysis will be conducted on the size and topography of incident ischemic and hemorrhagic strokes during the treatment period using clinical imaging obtained during the standard course of care by a core imaging laboratory at McMaster University.

**Table 3:** Study objectives and endpoints.

**Objectives**	**Endpoints**
**Primary**	
**Efficacy**	
To evaluate whether the oral FXIa inhibitor asundexian is superior to placebo on top of background antiplatelet therapy in reducing ischemic stroke in patients after an acute non-cardioembolic ischemic stroke or high-risk TIA	Ischemic stroke^a^
**Safety**	
To compare the incidence of ISTH major bleeding for asundexian and placebo on top of antiplatelet therapy in patients after an acute non-cardioembolic ischemic stroke or high-risk TIA	ISTH major bleeding^a^
**Secondary**	
**Efficacy**	
To evaluate whether asundexian is superior to placebo on top of antiplatelet therapy in reducing the occurrence of composite and individual efficacy endpoints	All strokes (ischemic and hemorrhagic)^a^ Composite of CV death, MI or stroke^a^ Composite of all-cause mortality, MI or stroke^a^ Ischemic stroke in the first 90 days^a^ Disabling stroke (mRS ≥3 or a 1-point increase if pre-stroke mRS if ≥) at 90 days^a^ All-cause mortality^a^ TIA^a^
**Safety**	
To compare asundexian and placebo on top of antiplatelet therapy with respect to individual bleeding endpoints	Composite of ISTH major or clinically relevant non-major bleeding^a^ ISTH clinically relevant non-major bleeding^a^ Symptomatic intracranial haemorrhage^a^ Hemorrhagic stroke^a^ Fatal bleeding^a^ Minor bleeding^a^
**Net clinical benefit**	
To further compare the benefit and risk of asundexian and placebo with respect to a composite of efficacy and safety endpoints	Composite of ischemic stroke or ISTH major bleeding^a^ Composite of CV death, all stroke, MI or ISTH major bleeding^a^ Composite of all-cause mortality, disabling stroke, fatal bleeding, symptomatic intracranial haemorrhage^a^
**Exploratory**	
**Efficacy**	
To further investigate the efficacy of the study intervention	Modified Rankin Scale Ischemic stroke after the first 90 days^a^
To investigate the effect of the study interventions on quality of life	EQ-5D questionnaire
**Safety**	
To further investigate the safety of the study intervention	Gastrointestinal bleeding^a^ BARC types 3 and 5 bleeding^a^ BARC types 2, 3 and 5 bleeding^a^ BARC type s1 and 2 bleeding^a^
**Net clinical benefit**	
To further compare the benefit and risk of asundexian and placebo with respect to hospitalisations	Total number of hospitalisations due to efficacy or safety outcome events
**Other exploratory (will be reported separately)**	
To further investigate the study intervention and drugs with similar, eg, mode-of-action-related effects, and to further investigate pathomechanisms deemed relevant to cardiovascular diseases and associated health problems	PK and various biomarkers (eg, diagnostic, safety, pharmacodynamic, monitoring, or potentially predictive biomarkers)

^a^Time to first occurrence.

Abbreviations: BARC = Bleeding Academic Research Consortium; CV = cardiovascular; EQ-5D = European Quality of Life Group 5 Dimension questionnaire; FXIa = Factor XIa; ISTH = International Society on Thrombosis and Haemostasis; MI = myocardial infarction; PK = pharmacokinetic(s).

An MRI substudy designed to explore the effect of asundexian on imaging markers of cerebrovascular disease enrolled approximately 1000 participants at selected sites and collected study specific images at baseline and 6 months post randomisation. Specifically, this substudy examines the effect of asundexian on incident infarcts, macrohaemorrhages, and cerebral microbleeds by comparing baseline and follow-up images. Images were read by trained readers blinded to treatment assignment at a corelab at the University of Calgary. Interested sites selected for feasibility of recruitment and imaging capability were qualified by submitting a phantom scan following a standard study protocol and were asked to scan at least 5 eligible consecutive OCEANIC-STROKE participants. Participants were considered eligible if they had no contraindications to MRI, had an index event of ischemic stroke, consented to the substudy, and could be scanned within 5 days of onset of symptoms.

### Sample size

OCEANIC-STROKE is an event-driven trial that is designed to continue until 830 participants have a new ischemic stroke confirmed by the adjudication committee. The planned sample size of 12,300 participants, 830 with events was increased from the initial size of 9,300 and 618 with events after a blinded review of the data showed that initial planning assumptions were not satisfied. In addition, the sample size increase allowed a greater power for secondary efficacy endpoints and treatment interactions in subgroups of interest. The number of participants was determined with the assumption of a placebo event rate of 6.3% at 12 months, a 2-sided type I error probability of 5% and 90% power for an effective (including effects of treatment discontinuation) hazard ratio (HR) of at least 0.80. With a minimum treatment duration of 90 days, we estimated a study duration of approximately 30 months until the CEOT period.

### Statistical analyses

Analysis of the primary outcome will be based on the intention to treat principle and include all randomised participants. Aalen-Johansen estimates of cumulative risk will be generated and HRs with 95% confidence intervals calculated. Aalen-Johansen estimates are preferred over Kaplan–Meier estimates as they account for the competing risk of death. Comparisons will be made using 2-sided stratified log-rank tests. Stratification will be by whether participants had a plan to receive dual or single antiplatelet therapy at randomisation. For secondary efficacy endpoints, the type I error rate will be controlled using a hierarchical testing procedure. The analyses of primary and secondary safety outcomes include all randomised participants who have taken at least one dose of the study intervention. All analyses will be detailed in the statistical analysis plan that will be finalised prior to database lock.

### Study organisation and funding

OCEANIC-STROKE was sponsored and funded by Bayer A.G. A Steering Committee comprised of the principal and co-principal investigators, national leaders, experts in stroke and thrombosis as well as sponsor representatives is supervising study conduct and are responsible for ensuring a high standard of scientific integrity. A Publications Committee oversees the quality and integrity of all publications and is composed of the principal and co-principal investigator, members of the Steering Committee and sponsor representatives.

An independent Data Safety Monitoring Committee (IDMC) composed of experts in stroke, thrombosis, clinical trials and statistics monitored the conduct of the study for safety and efficacy. An interim analysis for futility was performed after approximately 460 adjudicated ischemic strokes had occurred. The results of this analysis, conducted by an independent statistical group, were available only to the IDMC and were not shared with the Steering Committee, investigators or sponsor personnel.

### Role of the sponsor

The trial protocol was designed by the trial scientific leadership located at the Population Health Institute (PHRI), McMaster University with the sponsor. The Steering Committee provided input on key components of the protocol. The sponsor is responsible for trial operations including regulatory submissions, data collection, and site monitoring. Analyses for primary and secondary publications will be performed by study statisticians at PHRI. The sponsor may review proposed publications and may provide comments for accuracy, but sponsor approval is not required for submission. All publications will follow the ICJME criteria for authorship and Good Publication Practice.

## Results

Between January 2023 and February 2025, we randomised 12,327 participants from 703 sites in 37 countries. Table 4 provides demographic data as of 5 June 2025. The mean (SD) age was 67.6 (10.8) years and 4111 (33.3%) of participants were female. A previous history of stroke or TIA was noted in 2633 (21.4%) of participants with 1945 (15.8%) having coronary artery disease and 478 (3.9%) peripheral artery disease.

**Table 4:** Demographics as of 5 June 2025

**Randomised—no.**	12,327
**Age year—mean (SD)**	67.6 (10.8)
**Female sex—no. (%)**	4111 (33.3)
**Race—no. (%)**	
** White**	8185 (66.4)
** Black**	283 (2.3)
** Asian**	3450 (28.0)
** Other**	409 (3.3)
**Ethnicity—no. (%)**	
** Hispanic or Latino**	1031 (8.4)
** Not Hispanic or Latino**	10,918 (88.6)
** Not reported or Missing**	378 (3.1)
**Medical history—no. (%)**	
** Hypertension**	9780 (79.3)
** Diabetes**	4238 (34.4)
** Previous stroke or TIA**	2633 (21.4)
** Coronary artery disease**	1945 (15.8)
** Peripheral artery disease**	478 (3.9)
** Chronic kidney disease**	729 (5.9)
** Liver disease**	670 (5.4)
**Geographic Region—no. (%)**	
** North America**	1565 (12.7)
** South America**	567 (4.6)
** Western Europe, Australia and Israel**	4844 (39.3)
** Eastern Europe**	1823 (14.8)
** Asia**	3528 (28.6)
**Tobacco/nicotine use—no. (%)**	
** Never**	5739 (46.6)
** Former**	3276 (26.6)
** Current**	3309 (26.8)

Details of the index event are provided in Table 5. Most (94.7%) of participants entered the trial with an index event of ischemic stroke with the remainder entering with a high-risk TIA. The median (IQR) National Institutes of Health Stroke Scale score was 2 (1,4) at randomisation. Of those with ischemic stroke, a total of 3199 (27.4%) received hyperacute treatment with intravenous thrombolysis, endovascular therapy, or both prior to randomisation. Investigators classified the TOAST subtype as large artery atherosclerosis in 43.1%, undetermined aetiology in 30.0% and small-vessel occlusion in 22.2%. Most (62.6%) participants had a plan for DAPT with aspirin and a P2Y12 inhibitor at randomisation.

**Table 5:** Index event characteristics as of 5 June 2025

**Randomised—no.**	12,327
**Qualifying Event—no. (%)**	
**High-risk TIA**	649 (5.3)
** ABCD^2^ Score- median [IQR]**	6 [6,6]
**Ischemic stroke**	11,676 (94.7)
** TOAST subtype of index event—no. (%)**	
** Cardioembolism**	193 (1.7)
** Large-artery atherosclerosis**	5028 (43.1)
** Small-vessel occlusion**	2591 (22.2)
** Stroke of other aetiology**	358 (3.1)
** Stroke of undetermined aetiology**	3504 (30.0)
** Data missing**	2 (0.0)
** NIHSS at presentation**	
** median [min, max]**	3 [0,39]
** median [IQR]**	3 [2,6]
** NIHSS at randomisation**	
** median [min, max]**	2 [0,26]
** median [IQR]**	2 [1,4]
** Intravenous thrombolysis and/or endovascular therapy—no. (%)**	3199 (27.4)
** Intravenous thrombolysis only—no. (%)**	2312 (19.8)
** Endovascular therapy only—no. (%)**	371 (3.2)
** Intravenous thrombolysis and endovascular therapy no. (%)**	516 (4.4)
**Planned antiplatelet therapy at randomisation—no. (%)**	
** Dual (ASA and a P2Y12 inhibitor)**	7714 (62.6)
** Single antiplatelet therapy**	4613 (37.4)

## Discussion and conclusion

OCEANIC-STROKE will be the first completed superiority trial of FXIa inhibition for secondary stroke prevention. Asundexian added to standard of care antiplatelet treatment has the potential to decrease the risk of ischemic stroke following an initial ischemic stroke or high-risk TIA. Combined treatment appeared safe in phase 2 trials completed prior to beginning the current study. Randomisation of over 12,000 participants was completed in 2 years suggesting an interest in developing new antithrombotic options for stroke prevention, a pragmatic protocol and a robust global capacity for stroke prevention trials. The trial population includes commonly encountered stroke subtypes, a broad range of stroke severity, and participants who have received hyperacute treatment for the index event and should be readily generalisable. Trial completion is anticipated in October 2025 and holds the possibility of adding a new therapeutic class to the available antithrombotics for stroke prevention.

## Supplementary Material

aakaf017_OCEANIC-Stroke_Methods_Supplementary_Table_1

aakaf017_OCEANIC-Stroke_Methods_Supplementary_Table_2_clean

## Data Availability

Data from OCEANIC-STROKE will not be available until after completion of the trial. Details of availability will be provided in the primary results publication.
