# Supplementary material for: Rationale, design and baseline characteristics of participants in the OCEANIC-STROKE trial of FXIa inhibition for secondary stroke prevention
Source: Eur Stroke J. 2026 Jan 1;11(1):aakaf017. doi: 10.1093/esj/aakaf017 (PMC12866625; doi:10.1093/esj/aakaf017)
Supplement: aakaf017_OCEANIC-Stroke_Methods_Supplementary_Table_1 [file aakaf017_oceanic-stroke_methods_supplementary_table_1.docx]

**Supplementary Table 1.** *Primary* *efficacy estimand.*

|  |
| --- |
| **Definition of endpoint**  The primary efficacy endpoint is the time from randomization to the first occurrence of ischemic stroke.  **Main Analytical Approach**  In line with the while alive strategy applied to address the intercurrent event death for the primary efficacy estimand, death prior to the occurrence of a primary efficacy outcome will be considered a competing risk in the analysis. Addressing premature discontinuation of assigned treatment with the treatment policy strategy means that events occurring after treatment discontinuation are used in the analysis.  To estimate the relative change in the rate of the occurrence of the primary efficacy outcome in participants taking asundexian versus placebo according to the defined estimand, cause-specific hazard ratios (csHRs) and their associated confidence intervals will be derived from a stratified cause-specific Cox proportional hazards regression model. The results will be presented together with estimates of the csHRs for the associated competing risks.  The primary efficacy null hypothesis will be tested using a two-sided stratified log-rank test. |
|  |
