# Supplementary material for: Rationale, design and baseline characteristics of participants in the OCEANIC-STROKE trial of FXIa inhibition for secondary stroke prevention
Source: Eur Stroke J. 2026 Jan 1;11(1):aakaf017. doi: 10.1093/esj/aakaf017 (PMC12866625; doi:10.1093/esj/aakaf017)
Supplement: aakaf017_OCEANIC-Stroke_Methods_Supplementary_Table_2_clean [file aakaf017_oceanic-stroke_methods_supplementary_table_2_clean.docx]

**Supplementary Table 2.** *Primary safety estimand.*

|  |
| --- |
| **Main Analytical Approach**  In line with the “while on-treatment” strategy proposed to address intercurrent events for the primary safety estimand, both “death prior to the occurrence of a primary safety outcome” and “premature discontinuation of assigned treatment” will be considered competing risks in the analysis. This also implies that only participants who took at least one dose of study intervention will be included in the analysis.  To estimate the relative change in the rate of the occurrence of the primary safety outcome in participants taking asundexian versus placebo according to the defined estimand, csHRs and their associated confidence intervals will be derived from a stratified cause-specific Cox proportional hazards regression model. The results will be presented together with estimates of the csHRs for the associated competing risks.    If applicable, incomplete follow-up for the time to the primary safety outcome will be addressed with statistical methods aligned with the targeted estimand. |
